# Effects of multiple chronic conditions on health care costs: an analysis based on an advanced tree-based regression model

**DOI:** 10.1186/1472-6963-13-219

**Published:** 2013-06-15

**Authors:** Hans-Helmut König, Hanna Leicht, Horst Bickel, Angela Fuchs, Jochen Gensichen, Wolfgang Maier, Karola Mergenthal, Steffi Riedel-Heller, Ingmar Schäfer, Gerhard Schön, Siegfried Weyerer, Birgitt Wiese, Hendrik van den Bussche, Martin Scherer, Matthias Eckardt

**Affiliations:** 1 Department of Health Economics and Health Services Research, Hamburg Center for Health Economics, University Medical Center Hamburg-Eppendorf, Martinistraße 52, Hamburg, 20246, Germany; 2 Department of Psychiatry, Technical University of Munich, Ismaninger Straße 22, Munich, 81675, Germany; 3 Department of General Practice, University of Düsseldorf Medical Center, Moorenstraße 5, Düsseldorf, 40225, Germany; 4 Department of General Practice, Jena University Hospital, Bachstraße 18, 07743, Jena, Germany; 5 Department of Psychiatry and Psychotherapy, University of Bonn, Sigmund-Freud-Straße 25, Bonn, 53105, Germany; 6 Institute of General Practice, Goethe-University Frankfurt am Main, Theodor-Stern-Kai 7, Frankfurt am Main, 60590, Germany; 7 Institute of Social Medicine, Occupational Health and Public Health, University of Leipzig, Ph.-Rosenthal-Str. 55, Leipzig, 04103, Germany; 8 Department of Primary Medical Care, University Medical Center Hamburg-Eppendorf, Martinistraße 52, Hamburg, 20246, Germany; 9 Department of Medical Biometry and Epidemiology, University Medical Center Hamburg-Eppendorf, Martinistraße 52, Hamburg, 20246, Germany; 10 Central Institute of Mental Health, Medical Faculty Mannheim/Heidelberg University, Mannheim, D6, 68159, Germany; 11 Institute for Biometry, Hannover Medical School, Carl-Neuberg-Straße 1, Hannover, 30625, Germany

**Keywords:** Multiple chronic conditions, Multimorbidity, Co-morbidity, Health care costs, Conditional inference trees, Statistical learning

## Abstract

**Background:**

To analyze the impact of multimorbidity (MM) on health care costs taking into account data heterogeneity.

**Methods:**

Data come from a multicenter prospective cohort study of 1,050 randomly selected primary care patients aged 65 to 85 years suffering from MM in Germany. MM was defined as co-occurrence of ≥3 conditions from a list of 29 chronic diseases. A conditional inference tree (CTREE) algorithm was used to detect the underlying structure and most influential variables on costs of inpatient care, outpatient care, medications as well as formal and informal nursing care.

**Results:**

Irrespective of the number and combination of co-morbidities, a limited number of factors influential on costs were detected. Parkinson’s disease (PD) and cardiac insufficiency (CI) were the most influential variables for total costs. Compared to patients not suffering from any of the two conditions, PD increases predicted mean total costs 3.5-fold to approximately € 11,000 per 6 months, and CI two-fold to approximately € 6,100. The high total costs of PD are largely due to costs of nursing care. Costs of inpatient care were significantly influenced by cerebral ischemia/chronic stroke, whereas medication costs were associated with COPD, insomnia, PD and Diabetes. Except for costs of nursing care, socio-demographic variables did not significantly influence costs.

**Conclusions:**

Irrespective of any combination and number of co-occurring diseases, PD and CI appear to be most influential on total health care costs in elderly patients with MM, and only a limited number of factors significantly influenced cost.

**Trial registration:**

Current Controlled Trials ISRCTN89818205

## Background

The concept of multimorbidity (also referred to as multiple chronic conditions) relates to the coexistence of several chronic diseases in an individual [1]. Especially among the aged, multiple chronic conditions are common [2,3]. While there is no uniform cut-off point for multimorbidity, the coexistence of two or more and, alternatively, three or more chronic conditions are commonly used criteria [4]. In general, prevalence rates of multimorbidity among persons aged 65 and older have been widely reported to exceed 60% [5]. In a German study based on claims data from a large sample, van den Bussche et al. [6] found a prevalence of 62.1% for multimorbidity, defined as three or more conditions, among subjects aged 65 years or older and a mean number of 5.8 chronic conditions among these multimorbid subjects. As a result of demographic change, the prevalence of multimorbidity is expected to substantially increase in Germany and most other industrialized countries in the next decades.

Individuals with multiple chronic conditions consume a disproportionally large share of total health services. In a systematic literature review, Lehnert et al. [7] found ample evidence of a positive association between multimorbidity and health care costs. As a major result, the review reported that costs significantly increase with each additional chronic condition. Particularly physician visits, hospital use, and pharmaceuticals were found to elevate health care costs with each additional chronic condition. Yet the effect of additional chronic conditions on costs may depend on the number, type and combination of comorbidities with almost unlimited numbers of possible disease combinations. This heterogeneity can hardly be taken into account by traditional regression models.

This study aims to analyze the impact of multimorbidity on health care costs in Germany on all sectors of care. Instead of taking specific patterns of single disease combinations into account, our goal is to identify the most relevant diseases within arbitrary morbidity patterns influencing health care costs. Different from other studies, it tries to detect the underlying structure of cost data by using an improved tree-based graphical model. As a main advantage compared to traditional analytical methods, tree-based models allow to represent highly dimensional data in a simple manner and to easily interpret the results. Based on the method of automatic interaction detection (AID) introduced by Morgan and Sonquist [8], especially classification and regression trees (CART) have been widely used in health care research, including the analysis of comorbidity [9]–[11]. Nonetheless, CARTs tend to overfitting and a selection bias of covariates with a maximum number of possible splits. To overcome these weaknesses, advanced splitting algorithms like *Chi-square automatic interaction detectors* (CHAID) and conditional inference trees (CTREE) have been developed. As a limitation, CHAID requires categorical data and responses, while CTREE can deal with arbitrary scaled variables [12,13]. To our knowledge, this analysis is the first to use CTREEs for the analysis of cost data of multimorbid patients.

## Methods

### Sample and data

Data were collected within the MultiCare Cohort Study. Details regarding the methods of the study and the cohort have been published elsewhere [14]. The analyses presented here are based on data from the MultiCare baseline assessment. Briefly, the MultiCare Cohort Study is a multicenter, prospective cohort study of multimorbid primary care patients selected randomly from the databases of 158 general practitioners’ (GP) offices at 8 study centers across Germany. The study’s aims are to investigate multimorbidity patterns over time, to identify patients’ resources and risk factors that influence the course of these patterns, and to analyze the somatic, psychological and social consequences of these patterns for the patients’ quality of life and functional status. Inclusion criteria were age between 65 and 85 years, at least one visit to the GP within the last three-month period and multimorbidity, defined as the coexistence of at least 3 chronic conditions from a list of 29 conditions comprising alcohol-related disorders, anemia, anxiety disorders, atherosclerosis/peripheral artery occlusive disease (PAOD), asthma/chronic obstructive pulmonary disease (COPD), cancer, cardiac arrhythmias, cardiac insufficiency, cardiac valve disorders, cerebral ischemia/chronic stroke, chronic ischemic heart disease, depression, Diabetes mellitus, dizziness, intestinal diverticulosis, joint arthrosis, lower limb varicosis, migraine/chronic headache, neuropathies, osteoporosis, Parkinson’s disease, psoriasis, renal insufficiency, rheumatoid arthritis/chronic polyarthritis, severe hearing loss, severe vision reduction, somatoform disorders, thyroid dysfunction and urinary incontinence. Patients were excluded from the study if they were no regular patients of the GP. Other exclusion criteria were inability to participate due to medical reasons (such as blindness and deafness), insufficient German language skills, residence in a nursing home, and inability to provide informed consent or participation in another ongoing study. A diagnosis of dementia was therefore an exclusion criterion.

A total of 24,862 patients from the databases of the participating GP practices were checked for inclusion and exclusion criteria. 7,172 patients fulfilled the criteria and were contacted for informed consent to participate. 3,317 patients agreed to participate and were available for the baseline interview within a time frame of 16 months. In retrospect, 128 of these cases had to be excluded either because, in direct contact, exclusion criteria were found to apply or because the patient died before the baseline interview. Thus, a final number of 3,189 patients were included in the study.

Of these, 1,051 patients (i.e. an approximate third of the cohort) were randomized into a subsample in which a comprehensive assessment of healthcare resource use was conducted in addition to the standard MultiCare assessment battery. Due to a missing value for health insurance, one case was excluded from this subpopulation. Thus, the analyses presented here are based on this subsample of N = 1050. Recruitment and baseline interviews took place between July 2008 and October 2009. The study was approved by the Ethics Committee of the Medical Association of Hamburg.

### Multimorbidity

Multimorbidity was assessed by means of a standardized GP questionnaire which comprised 46 chronic conditions including the 29 conditions used as inclusion criteria. The list was newly compiled at the beginning of the MultiCare study with the aim of representing the most frequent chronic conditions in the population and is based on prevalence data (for details, see [6,14]). Yet in order to ensure a wide range of diseases and syndromes, those with a prevalence of more than 25% were not used as inclusion criteria for the sample, as an unselected application of the three-condition-criterion would have resulted in an overrepresentation of these very frequent diseases and a small number of disease patterns in the study population [14]. Nevertheless, these highly prevalent conditions are frequently combined with the relatively lower prevalent ones and therefore still part of the sample.

In the 46 conditions, ICD-10 codes are classified together if diseases and syndromes are similar pathophysiologically or if ICD-codes of related disorders are used ambiguously in practice. At the beginning of the baseline interviews, the compilation of the list had not been quite finalized, and for this reason 7 of the conditions were not part of the standardized baseline GP questionnaire, but were assessed by means of open questions. This applies to chronic gastritis, insomnia, allergies, obesity, hypotension, sexual dysfunction and tobacco abuse. The conditions assessed in a standardized fashion in the GP questionnaire at baseline comprise the 29 condition used as inclusion criteria as well as chronic cholecystitis/gallstones, chronic low back pain, haemorrhoids, hypertension, lipid metabolism disorders, liver diseases, noninflammatory gynaecological problems, purine/pyrimidine metabolism disorders/gout, prostatic hyperplasia and urinary tract calculi. Dementia was also listed, but constituted an exclusion criterion at baseline.

### Sociodemographic variables

Socio-economic status (education, income) was assessed with an established questionnaire [15]. The level of education was rated according to the international CASMIN classification [16]. Income is reported as net monthly income from all sources of income adjusted for household size (this is net income divided by the equivalized household size, for which a value of 1.0 is assigned to the householder, 0.5 is assigned to every other household member aged 15 or over, and 0.3 is assigned to every child under the age of 15).

### Resource use

Resource use was recorded by means of a questionnaire administered as part of the MultiCare assessment battery. The resource use questionnaire was developed by our working group. It is based on versions used in previous investigations (e.g., [17]–[19]) and is available from the authors upon request. The questionnaire covers in patient treatment, out-patient physician treatment, pharmaceuticals, other kinds of out patient treatment (such as physical or occupational therapy), medical supplies and dental prostheses, nursing home care, professional nursing services and other paid help as well as informal care (Table 1). The items for informal care are based on an instrument by Neubauer et al. [20]. Assessment was retrospective and covered a period of 3 months, except for in patient treatment and nursinghome care, for which the period was 6 months. The questionnaire contains lists of common resources and services in order to minimize recall bias.

**Table 1:** Documented resource use and unit costs applied for calculation of costs

**Sector**	**Services / Goods**	**Units**	**Unit costs (Source)**
Inpatient treatment	Stays in general hospitals, specialized psychiatric and neurological hospitals or rehabilitation clinics (including day-patient treatment)	Days in hospital	Per diem costs by type (Federal Statistical Office, German Hospital Federation, Statutory Pension Insurance Fund [34]–[36])
Outpatient physician treatment	Treatment by GPs, specialists and outpatient clinics	Number of contacts	Calculated costs per contact, by specialization [37]
Other outpatient treatment	E.g., physiotherapy, massage, occupational therapy, speech therapy	Number of contacts	Reimbursement schedules (Statutory health insurance funds [38]–[40]), calculated costs per contact [37], by type
Medical supplies and dental prostheses	E.g., walkers, incontinence pads, hearing aids, surgical stockings; bridge, crown	Quantity	Reimbursement schedules (Statutory health insurance funds, Federal Association of Panel Dentists[41,42]), calculated costs per item [37], by type
Medication	Specific products (including trade name, drug code, package size, pharmaceutical form, dosage)	Quantity	Pharmacy retail prices (Rote Liste 2008 [43])
Nursing home care	Nursing home stays (residential and day care)	Days	Calculated costs of care per day (Federal Statistical Office [44]), by type
Professional home care	Care and assistance provided by professional nursing services and other paid help, differentiated by type (e.g., basic care, assistance with cleaning, shopping, financial matters etc.) and limited to care or assistance required due to illness or age	Hours	Hourly gross wage rate plus non-wage labor costs for employees in the domain of care and assistance for the elderly or handicapped (Federal Statistical Office [45,46])
Informal care	Care and assistance provided by family or friends, differentiated by type and limited to care or assistance required due to illness or age	Hours	Replacement cost method: Hourly gross wage rate plus non-wage labor costs for employees in the domain of care and assistance for the elderly or handicapped (Federal Statistical Office [45,46])

GP = General practitioner.

### Health care costs

We adopted a societal perspective; therefore all resources and services used were recorded, regardless of whether they were covered by health or nursing insurance or paid for out-of-pocket. The cost categories analyzed in this study are direct costs of illness arising from the use of resources. We did not evaluate indirect costs due to lost productivity because of the advanced age of the subjects. Healthcare costs were calculated for a 6-month period, multiplying resource use by two in sections which covered a 3-month period. Costs were calculated from resource use as recorded in the questionnaire by means of unit costs. Resource categories and sources of unit costs are listed in Table 1. Informal care was valued using the replacement cost approach, i.e. it was assumed that the same amount of care by professional nursing services would have had to be paid for in the absence of an informal caregiver. Accordingly, hours of informal care were valued using the same hourly wage rate as for professional home care (see van den Berg [21] for an overview of methods for the valuation of informal care).

Cost were calculated in € at 2009 price levels. Unit costs that were unavailable at year 2009 values were inflated or deflated to year 2009 price levels by means of the consumer price index [22].

For statistical analysis, we categorized cost data as follows: 1) Costs of inpatient care comprising inpatient treatment in general hospitals, specialized psychiatric and neurological hospitals or rehabilitation clinics; 2) costs of outpatient care comprising outpatient physician treatment, other outpatient treatment, and medical supplies and dental prostheses; 3) costs of medication comprising pharmaceuticals; 4) costs of nursing care comprising nursing home care, professional home care and informal care.

### Missing values

Missing values in items of the resource use questionnaire (below 1% for all items) were imputed using the means of the observed data for the respective items (conditional means). Dosage of medication was an exception, however, since medications and their dosage were too varied interindividually for mean imputation to be possible. Therefore costs for medication with missing values for dosage were calculated using a conservative rule, whereby the pharmacy retail price of one package of the drug per 3 months was applied. Missing values in items of the standard MultiCare assessment battery were imputed using the hot deck method, in which missing values are replaced using observed values from a responding unit that is as similar as possible to the non-responding unit [23,24]. The proportion of missing values in those items which were used in our analysis was below 0.3%, except for income with 12.7% missings.

### Statistical analyses

We used a conditional model based on a supervised learning tree-algorithm in order to visualize a hierarchical data partition and to detect the underlying structure and most influential variables on total costs and on costs of the four different cost sectors (inpatient care, outpatient care, medication, nursing care) separately. As covariates we used binary variables for those 41 of the 46 diseases which had a prevalence rate of ≥1% in the sample. In addition, we included a binary variable for obesity (defined as body mass index ≥ 30 kg/m^2^). A detailed list of the diseases taken into account is provided in the Results section. Additionally, female sex (reference category: male) and private health insurance (reference category: statutory health insurance) were added as dichotomous variables. Education was included as a categorical variable, taking low education as reference. Besides this, age and the logarithmic (log) transformed income were included as continuous covariates. Log-transformation was used to achieve a linear relationship of predictors and outcomes. No additional distributional assumptions concerning the error terms or outcomes were made.

Traditionally, classification and regression trees (CART) attempt to discriminate data into homogenous subsets. Thus, node *A* is split into two disjunctive subsets *A* ∩ {*x*_*i*_ ≤ *c*} and *A* ∩ {*x*_*i*_ > *c*} based on a single variable *X*_*i*_ = *x*_*i*_ (see [25]). As splitting criteria, the impurity of node or entropy could be used. One main disadvantage of CARTs is their tendency to grow huge trees by selecting splitting variables which lead to a maximum tree size. As an attempt to reduce the tree size, the optimal cut subtree could be detected using minimal cost-complexity pruning based on cross validation (see [26]).

To overcome these disadvantages and as a superior approach to CART, we used in a first step a non-parametric conditional inference tree (CTREE)-algorithm predicated on recursive binary partitioning embedded in the framework of permutation tests introduced by Strasser and Weber [27]. Thus the distribution of the response *Y* is defined as conditional on a function *g* of a set of *k* arbitrarily scaled covariates *X* as *f*(*Y*|*g*(*X*_1_, …, *X*_*k*_)).

A learning sample *L*_*n*_ based on a random selection of *n* i.i.d. observations was used to fit the tree-structured regression model. A vector of dichotomous case weights *w* representing each node was used to create the disjunctive subsets *w*_*left*,*i*_ = *w*_*i*_*I*(*X*_*i*_ ∈ *A*) and *w*_*right*,*i*_ = *w*_*i*_*I*(*X*_*i*_ ∉ *A*) with *I*(⋅) as indicator function. A discrepancy measure of the form

$$T_{j}\left(L_{n},w\right)=\mathrm{vec}(\sum_{i=1}^{n}w_{i}I\left(X_{\mathrm{ij}}\right)h(Y_{i},{\left(Y_{1},\ldots ,Y_{n})\right)}^{T})$$

was used to establish a two sample statistic for all possible subsets of *A* with *h*(⋅) as influence function. *vec* is the vec-operator and (⋅)^*T*^ the transpose.

At each node a global null hypothesis *H*_0_ : *f*(*Y*|*X*_*j*_) = *f*(*Y*) was tested on a pre-specified *α* = 0.05 level of significance. To incorporate different scaled covariates, a maximization of the test statistic based on the conditional mean and conditional variance over all possible subsets was established. In case of acceptance, the tree-algorithm was interrupted and no further data-split was performed. Otherwise the covariate *X*_*j*_ with the strongest influence on *Y* was selected as node, and the null hypothesis was tested in each subset of the tree. This approach guarantees the optimal sized tree is grown [12,28]. To visualize the inherent structure, trees were plotted for total costs and each cost sector.

The cost means ${\hat{\mu }}_{i}$ were predicted with regard to the number of case weights *w*_*i*_ = 1. To evaluate the prediction quality, both the squared error loss and the mean absolute error were calculated.

In addition, in order to evaluate model performance of CTREE, it was compared to traditional CART, which is an alternative tree-based algorithm.

In a second step we used ensemble methods in terms of conditional random forests. *n*_*TREE*_ = 500 random trees were grown to increase the performance of our predictions and to verify our results [25]. As a benefit, especially random forests can deal with large covariate lists and/or complex interaction structures. In contrast to random forests introduced by Breimann [29], which are based on CART, we implemented an unbiased random forest based on CTREE [30]. Based on the unbiased random forest variance, importance scores were calculated indicating the importance of certain variables for determining the response. Basically, variable importance measures the difference in prediction accuracy before and after randomly permuting single covariates.

The analysis was performed using the packages party and rpart in R 2.14.1 [31].

## Results

### Sociodemographic and morbidity data

The mean age of the sample at baseline was 74.4 years, and 58.6% were female (Table 2). More than half of the participants were married (56.9%), while approximately one quarter were widowed (27.5%). 58.3% were living with their spouse or partner, and 35.2% were living alone in their own home. The proportions of subjects in assisted living (2.0%) or retirement homes (0.3%) were low. The majority of the sample had a low degree of education (61.8%), and mean household-size adjusted monthly income was € 1,440 . Only 4.3% of the participants were privately insured.

**Table 2:** Characteristics of the sample (N = 1,050)

	
Age: mean (SD)	74.4 (5.20) years
Gender: N (%) female	616 (58.67)
Marital status: N (%)	
Married	598 (56.89)
Married, but separated	26 (2.47)
Single	68 (6.47)
Divorced	69 (6.56)
Widowed	289 (27.49)
Living situation: N (%)	
Alone	370 (35.20)
With spouse/partner	613 (58.32)
With family members	40 (3.81)
With others	3 (0.29)
Assisted living	21 (2.00)
Retirement home	3 (0.29)
Education: N (%)	
Low	649 (61.81)
Medium	297 (28.29)
High	104 (0.10)
Income: mean (SD)	€ 1,440 (€ 737)
Number of chronic conditions (SD)	7.0 (2.50)
Type of health insurance: N (%)	
private	45 (4.29)
statutory	1,005 (95.71)

On average, the participants had 7.0 chronic conditions, with no significant differences between men and women. The ten most prevalent conditions in the overall sample, in descending order, were hypertension (79.4%), lipid metabolism disorders (59.4%), chronic low back pain (51.0%), joint arthrosis (43.4%), Diabetes mellitus (38.2%), chronic ischemic heart disease (32.7%), obesity (31.3%), thyroid dysfunction (31.0%), cardiac arrythmias (28.5%) and osteoporosis (26.4%) (Table 3). However, there were some differences in rank order by gender. For instance, the prevalence of chronic ischemic heart disease was twice as high for men (43.3%) as for women (25.2%), for whom this condition only ranked eleventh. Osteoporosis, by contrast, was much more common among women, for whom this condition ranked eighth, than among men (30.7% vs. 5.8%). Among men thyroid dysfunction and lower limb varicosis were much less common than among women (20.1% vs. 38.8% and 14.3% vs. 29.4%), while the ten most prevalent conditions for men also included prostatic hyperplasia (28.3%, ranking seventh) and purine/pyrimidine metabolism disorders and gout (27.2%, ranking ninth). More details on the prevalence of chronic conditions in the MultiCare cohort have been reported elsewhere (see [32]).

**Table 3:** Prevalence of chronic conditions and rank order in the sample, overall and by gender

**Chronic condition**	**Prevalence in % (rank)**		
	**Overall n = 1,050**	**Female n = 616**	**Male n = 434**
Hypertension	79.4% (1)	79.5% (1)	79.3% (1)
Lipid metabolism disorders	59.4% (2)	57.5% (2)	62.2% (2)
Chronic low back pain	51.0% (3)	57.4% (3)	42.2% (5)
Joint arthrosis	43.4% (4)	50.2% (4)	33.9% (6)
Diabetes mellitus	38.2% (5)	34.4% (7)	43.5% (3)
Chronic ischemic heart disease	32.7% (6)	25.2% (11)	43.3% (4)
Obesity	31.3% (7)	35.6% (6)	25.3% (11)
Thyroid dysfunction	31.0% (8)	38.8% (5)	20.1% (14)
Cardiac arrhythmias	28.5% (9)	26.5% (10)	31.3% (7)
Osteoporosis	26.4% (10)	30.7% (8)	5.8% (33)
Asthma/COPD	23.5% (11)	20.9% (13)	27.2% (9)
Lower limb varicosis	23.1% (12)	29.4% (9)	14.3% (20.5)
Purine/pyrimidine metabolism disorders/Gout	20.2% (13)	15.3% (16.5)	27.2% (10)
Severe vision reduction	19.0% (14)	18.5% (14)	19.8% (15)
Cancer	18.1% (15)	15.3% (16.5)	22.1% (13)
Depression	17.3% (16)	21.9% (12)	10.8% (24)
Atherosclerosis/PAOD	17.2% (17)	12.7% (18)	23.7% (12)
Intestinal diverticulosis	15.4% (18)	16.1% (15)	14.5% (19)
Neuropathies	13.7% (19)	11.0% (20.5)	17.5% (16)
Chronic gastritis/GERD	13.1% (20)	12.3% (19)	14.3% (20.5)
Cardiac insufficiency	12.6% (21)	11.0% (20.5)	14.8% (18)
Renal insufficiency	12.4% (22)	9.6% (24)	16.5% (17)
Prostatic hyperplasia	11.7% (23)	-	28.3% (8)
Cerebral ischemia/Chronic stroke	11.2% (24)	9.7% (22)	13.3% (22)
Cardiac valve disorders	9.1% (25)	8.9% (27)	9.5% (26)
Haemorrhoids	9.0% (26)	5.4% (31.5)	12.7% (23)
Dizziness	8.8% (27)	9.6% (24)	7.7% (28)
Chronic cholecystitis/Gallstones	8.1% (28)	8.0% (28)	8.3% (27)
Liver diseases	7.9% (29)	6.3% (29)	10.1% (25)
Somatoform disorders	7.4% (30)	9.1% (26)	5.1% (34)
Urinary incontinence	7.3% (31)	9.6% (24)	4.2% (35)
Insomnia	5.0% (32)	4.2% (35)	6.2% (31)
Severe hearing loss	4.7% (33)	3.4% (37)	6.4% (29)
Anemia	4.6% (34)	3.2% (38)	6.5% (30)
Psoriasis	4.2% (35)	2.9% (39)	6.0% (32)
Rheumatoid arthritis/Chronic polyarthritis	4.0% (36)	5.5% (30)	1.3% (40)
Anxiety	3.7% (37)	4.6% (33.5)	2.5% (37)
Allergies	3.6% (38)	4.6% (33.5)	2.3% (38.5)
Migraine/chronic headache	3.5% (39)	5.4% (31.5)	0.9% (41)
Parkinson’s disease	2.3% (40)	2.0% (40)	2.8% (36)
Noninflammatory gynaecological problems	2.1% (41)	3.6% (36)	-
Urinary tract calculi	1.5% (42)	1.0% (41)	2.3% (38.5)

COPD = Chronic obstructive pulmonary disease; GERD = Gastroesophageal reflux disease; PAOD = Peripheral artery occlusive disease.

### Costs

In this section we present the results of the conditional inference trees. At first we report on the analysis of total costs, followed by costs of inpatient care, outpatient care, medication and nursing care. All cost data refer to a 6-month period.

#### Total costs

**Figure 1:**
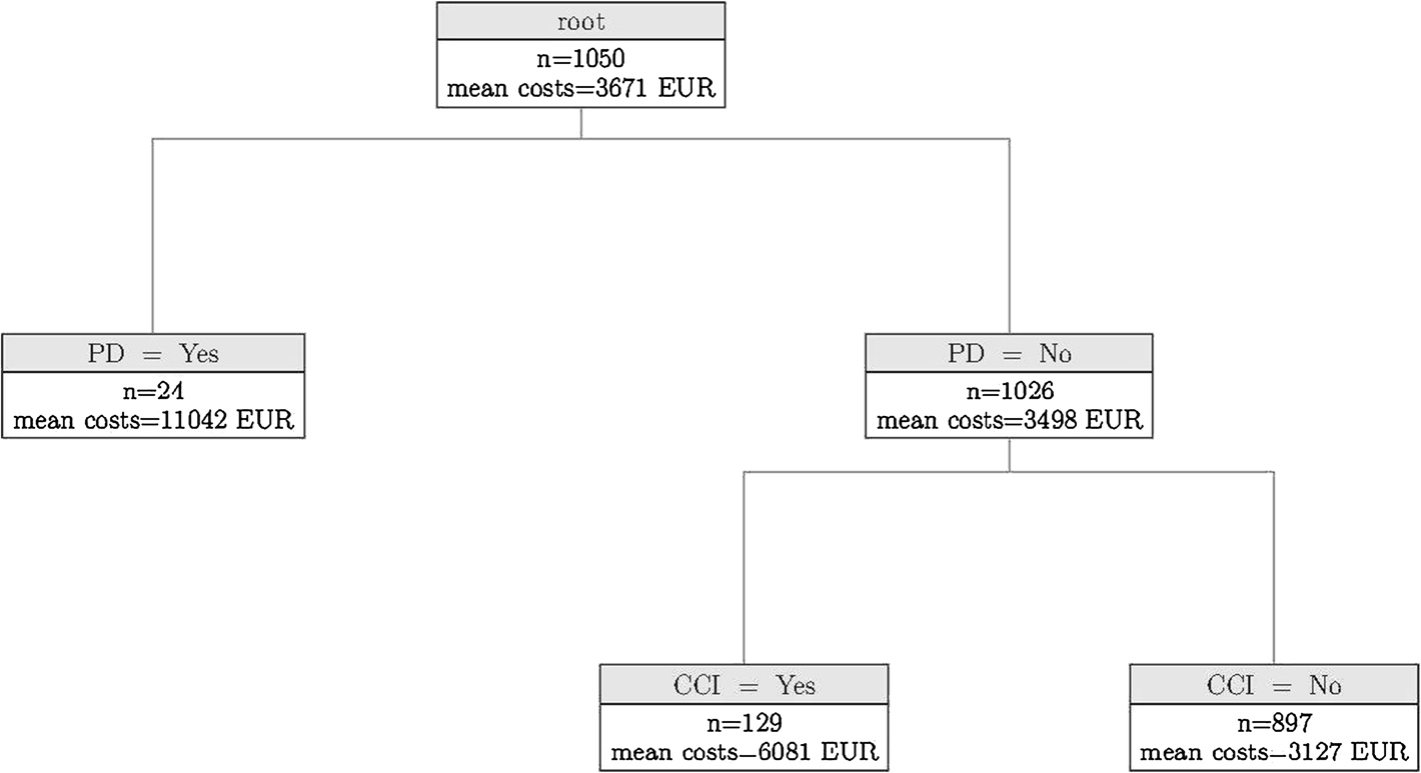
**Conditional independence tree for total costs.** PD = Parkinson’s disease; CCI = cardiac insufficiency; mean costs = predicted mean total costs in € in 6-month period.

Mean total costs in the whole sample amounted to € 3,671 (SD: € 6,996), ranging from € 23 to € 101,600. The identified tree model consisted of 5 nodes defining three homogenous subsets based on two dichotomous disease indicators (Figure 1). The first split was caused by the covariate Parkinson's disease (PD) at a significance level of p < 0.001. Given that PD occurs within the individual multimorbidity pattern, the model predicts mean total costs of € 11,042 (n = 24, SD: € 14,216) with no further split. If PD is not present, a further split is caused by another disease covariate indicating the presence of cardiac insufficiency (p < 0.001). Conditional on the absence of PD, predicted mean costs are € 6,081 (n = 129, SD: € 11,498) if cardiac insufficiency is present, and € 3,127 (n = 897, SD: € 5,535) if not. Thus, regardless of any other variables taken into account or co-existing combinations of diseases, total costs are influenced by the presence or absence of PD and cardiac insufficiency.

#### Costs of inpatient care

**Figure 2:**
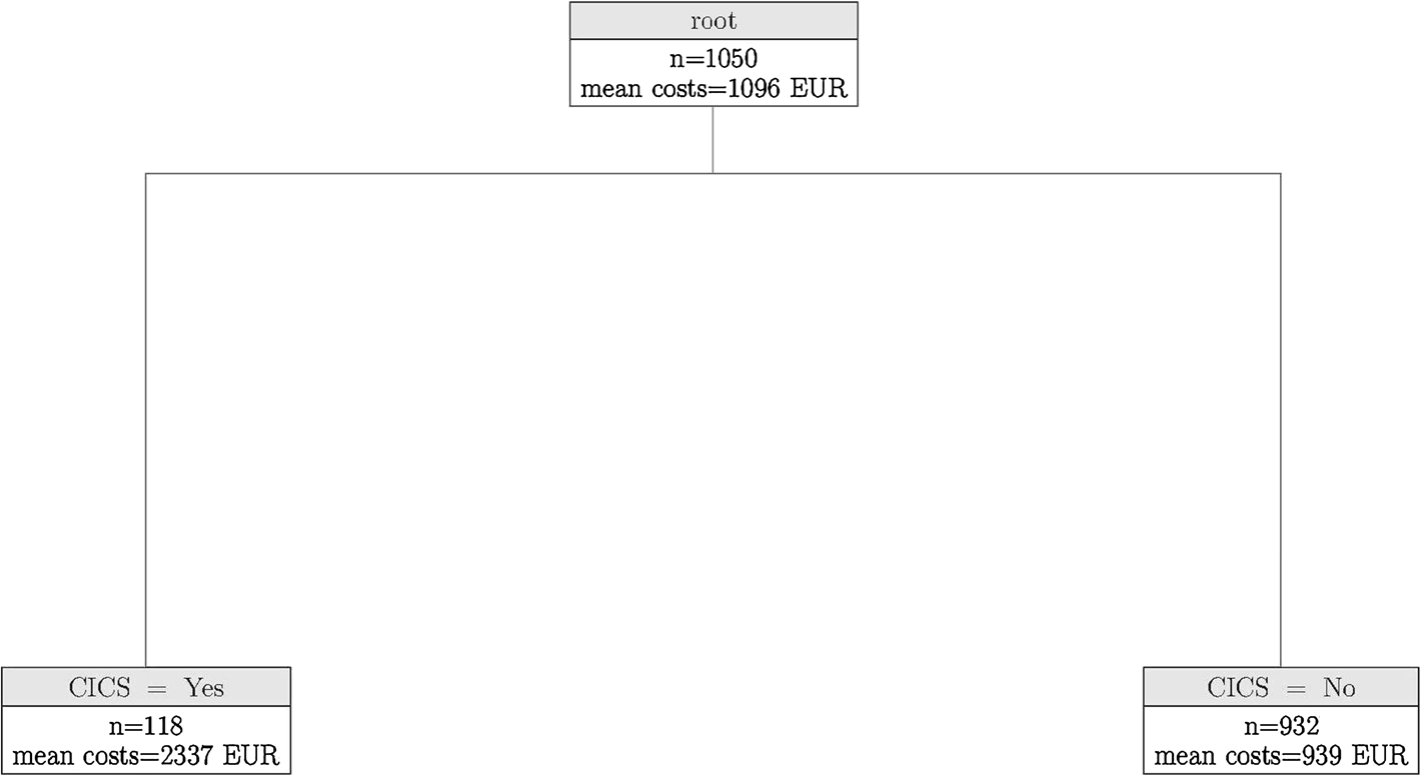
**Conditional independence tree for inpatient costs.** CICS = cerebral ischemia and/or chronic stroke, mean costs = predicted mean inpatient costs in € in 6-month period.

Mean costs of inpatient care in the whole sample amounted to € 1,096 (SD: € 4,029), ranging from € 0 to € 92,850. For inpatient care we identified a tree-based model consisting of only 3 nodes (Figure 2): the only split was caused by cerebral ischemia and/or chronic stroke (CI/CS) at a significantly level of p = 0.018. Predicted mean hospital costs are € 2,337 if CI/CS is present (n = 118, SD: € 9,373), and € 939 otherwise (n = 932, SD: € 2,653).

#### Costs of outpatient care

Mean costs of outpatient care in the whole sample amounted to € 418 (SD: € 846), ranging from € 0 to € 25,120. For outpatient costs no split was detected at the *α* =0.05 level of significance. When increasing the significance level α to 0.2, a single split was achieved by cardiac insufficiency (p = 0.072).

#### Costs of medication

**Figure 3:**
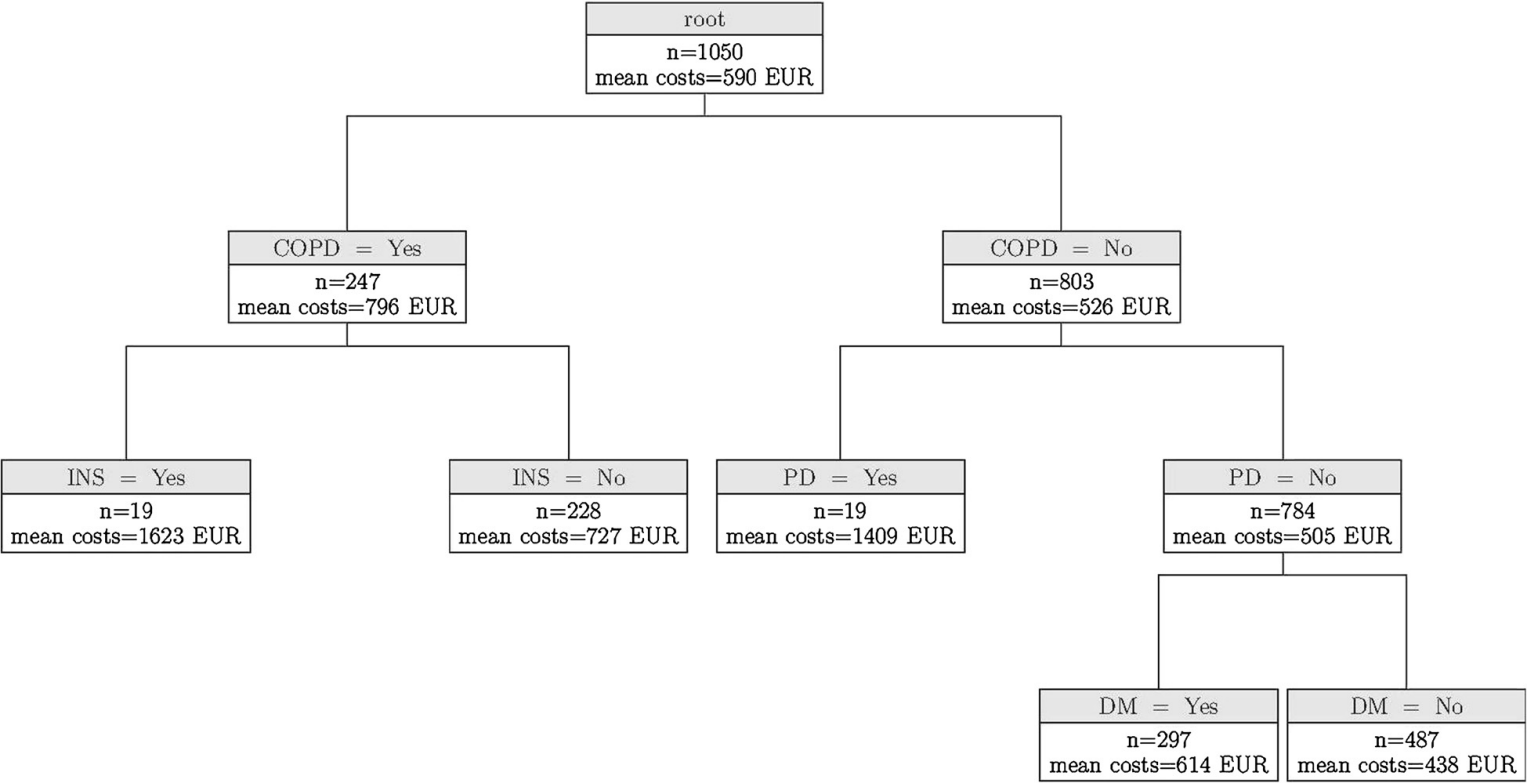
**Conditional independence tree for medication costs.** COPD = chronic obstructive pulmonary disease; PD = Parkinson’s disease; INS = insomnia; DM = Diabetes mellitus, mean costs = predicted mean medication costs in € in 6-month period.

Mean costs of medication in the whole sample amounted to € 590 (SD: € 752), ranging from € 0 to € 15,440. With respect to medication costs, nine nodes were identified with four chronic conditions influencing costs (Figure 3). The first split was caused by chronic obstructive pulmonary disease (COPD), which has a highly significant (p < 0.001) impact on the medication costs. Given that COPD is present, a further split is caused by insomnia (p < 0.032). If COPD is present, mean predicted medication costs amount to € 1,623 if insomnia is also present (n = 19, SD: € 3,400) and € 727 if not (n = 228, SD: € 587). On the other hand, if there is no diagnosis of COPD, PD causes a further split (p < 0.001) with predicted mean medication costs of € 1,409 if PD is present (n = 19, SD: € 1,510). If PD is not present, a further split is caused by Diabetes mellitus (p < 0.001), with predicted mean costs of € 614 (n = 297, SD: € 591) if present and € 438 (n = 487, SD: € 487) if not.

#### Costs of nursing care

**Figure 4:**
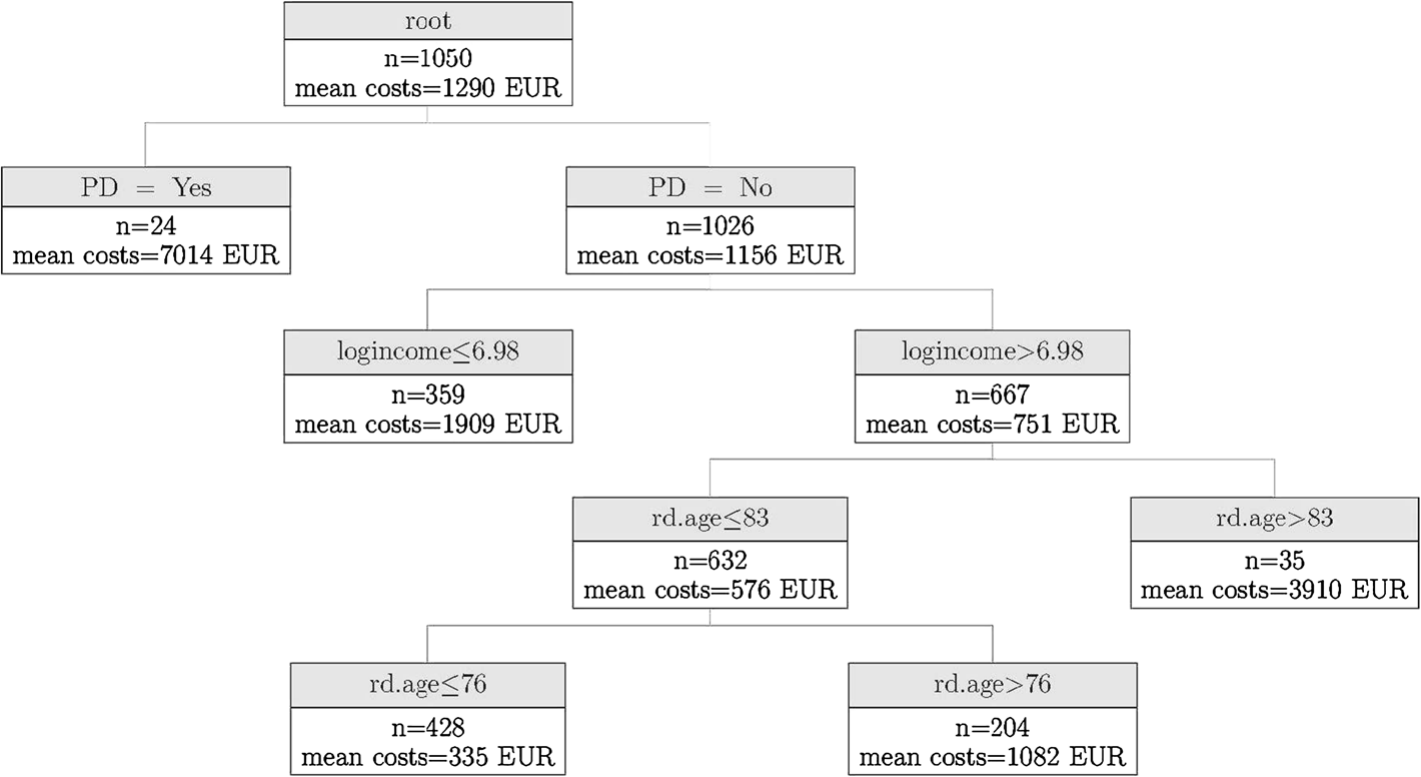
**Conditional independence tree for costs of nursing care.** PD = Parkinson’s disease; rd age = rounded age; logincome = natural logarithm of income, mean costs = predicted mean costs of nursing care in € in 6-month period.

Mean costs of formal and informal nursing care in the whole sample amounted to € 1,290 (SD: € 4,815), ranging from € 0 to € 50,040. For costs of nursing care, nine nodes were identified (Figure 4). The first split was caused by PD, which has a highly significant impact on costs of nursing care (p < 0.001). If PD is present, our model predicts mean nursing care costs of € 7,014 (n = 24, SD: € 13,475). Given that PD is not present, a further split is caused by the logarithmic income ≤ € 6.98 (e^€6.98^≈ € 1,075) or > € 6.98 (p = 0.015). Given that logarithmic income is ≤€ 6.98, the predicted mean nursing care costs are € 1,909 (n = 359, SD: € 5,973). If the income is higher than that, a further split is caused by age, with three age groups being detected: at a first step a split is detected at >83 years (p < 0.001), leading to predicted mean nursing care costs of € 3,910 (n = 35, SD: € 8,278). In case of an age ≤83 years, a further split is caused by an age >76 years (p = 0.006), leading to predicted mean care costs of € 1,082 (n = 204, SD: € 3,418). Alternatively, given an age below 76 years, mean nursing care costs of € 335 (n = 428, SD: € 1,617) are predicted.

### Conditional random forests and comparison with CART

Based on a conditional random forest consisting of 500 conditional random trees, variance importance scores were calculated to verify our findings. As a main result, the numerical order of these scores coincide with the structure described above throughout all sectors. Thus, we were able to confirm the accuracy of our analysis using ensemble methods. Independently of our specific sample, we detected the factors most influential on costs irrespective of the number and combination of co-occuring comorbidities.

For comparison with CTREE, classification and regression trees were computed for all cost sectors. As expected, CARTs lead to bigger grown trees. In particular, in the case of nursing care 19 nodes emerged, resulting in 10 subsets including as splitting criteria PD, logarithmic income, Diabetes mellitus, cerebral ischemia/chronic stroke, age, liver diseases and cancers. At the same time, similar to the CTREE algorithm, the first split was caused by PD. With respect to total cost, 11 nodes resulted using CART, including PD, cardiac insufficiency, cerebral ischemia/chronic stroke, osteoporosis and renal insufficiency. Thus, CART included three additional diseases compared to CTREE. In case of medication costs, 7 nodes were identified by CART consisting of PD, asthma/chronic obstructive pulmonary disease and logarithmic income. Furthermore, opposite to CTREE, CART resulted in 5 nodes for outpatient care. These include logarithmic income and osteoporosis as splitting variables. Finally, 9 nodes emerged for costs of inpatient care, including cerebral ischemia/chronic stroke, neuropathies, urinary incontinence and cardiac arrhythmia as split variables. Compared to CTREE, only cerebral ischemia/chronic stroke was used as nodes in both tree algorithms.

In order to control for overfitting and finding the optimal CART based tree size, minimal cost-complexity pruning was applied. This method was not able to find any suitable tree in any cost sector.

Mean absolute errors of CTREEs and the “not pruned” CARTs are reported in Table 4. For costs of outpatient care and costs of nursing care, CTREE achieved a lower mean absolute error value compared to CART. However, with respect to total costs, costs of medication and costs of inpatient care CART lead to smaller error terms. All splitting variables of CTREE were used as splitting variable of the trees grown by CART.

**Table 4:** Comparison of absolute mean error of CTREE and CART algorithms for different cost sectors

**Cost sector**	**Mean absolute error**	
	**CTREE**	**CART**
Total costs	3,690.87	3,611.64
Outpatient care	284.09	293.39
Inpatient care	1,761.90	1,709.78
Medication	2,012.37	1,919.07
Nursing care	388.12	398.37

CART = classification and regression tree; CTREE = conditional inference tree.

## Discussion

The partitioning conditional tree algorithm allows to detect the underlying structure of how certain diseases within arbitrary multimorbidity patterns influence the costs of health care. Using this statistical approach, we found various variables which are associated with total costs, inpatient costs, medication costs and nursing care costs in multimorbid elderly. These results were verified using ensemble methods.

With respect to total costs and independent from the other co-existing comorbidities, PD and cardiac insufficiency were identified as the most influencial variables, with PD being the more important one. Compared to patients not suffering from any of the two conditions, PD increases predicted mean total costs 3.5-fold to approximately € 11,000 per 6 months, and cardiac insufficiency 2-fold to approximately € 6,100.

The high total costs of PD are largely due to costs of nursing care, for which the respective partitioning tree model predicted more than € 7,000 on average in this patient group. When excluding nursing care from total costs, PD disappeared in the tree for total costs, while the split from cardiac insufficiency remained significant (p = 0.004), predicting mean total costs of € 3,790 (n = 132) if cardiac insufficiency is present and € 2,177 (n = 918) otherwise (tree not shown). The same reduced tree structure resulted when only excluding costs of informal nursing care from total cost, predicting mean total costs of € 4,052 if cardiac insufficiency is present and € 2,260 otherwise (p = 0.001; tree not shown), and reflecting that high nursing costs of PD are largely due to informal care.

If PD is not present, mean nursing care costs are influenced by income and age, with low income being associated with higher costs and, in those with higher income, age being associated with higher costs. Taking comparatively more affluent patients aged ≤76 years not suffering from PD as the reference group, patients with similar income in the age groups 77–83 and >83 cause more than 3-fold and almost 11-fold mean nursing costs, respectively, if PD is not present. If PD is present, mean nursing costs are elevated almost 21-fold compared to the same reference group, irrespective of age and income. In patients with comparatively low income without PD, mean nursing costs are increased almost 5-fold compared to the reference group irrespective of age.

PD was also found to increase medication costs. Yet concerning medication costs, the coexistence of COPD and insomnia was identified as being associated with the highest mean medication costs. Besides these conditions, Diabetes mellitus significantly increases medication costs if COPD and PD are not present. Compared to patients in whom neither Diabetes nor PD or COPD are present, Diabetes (without PD) increases mean medication costs by 40%, PD by 222%, and COPD by 66% or even 271% if insomnia is also present.

While the partitioning tree algorithm identified no variable significantly associated with outpatient costs, cerebral ischemia and/or chronic stroke (CI/CS) was found to increase inpatient costs 3.5-fold, with no other variables being significant in the model.

Except for costs of nursing care, socio-demographic variables did not significantly influence costs of care.

### Strengths and limitations

In general, one main advantage concerning partitioning tree algorithms compared to traditional analytical methods is to be seen in the simplified representation of high dimensional data and its direct interpretability. Due to the chosen 0/1 recursive partitioning framework, the lack of smoothness - a common disadvantage of tree based modelling - could be neglected.

Compared to classical parametric regression techniques, tree-based decision models avoid any distributional assumption. Therefore, the estimation of the coefficients is not affected by misspecification. At the same time, trees aim to discriminate disjunctive homogeneous subsets by minimizing within-variance and maximizing between-variance.

As a main disadvantage, CART or related decision tree algorithms like C4.5 face high variance caused by the inherent binary partitioning method, leading to a propagation of the error effect of the first split. Besides this, due to their focus on the maximization of the information criteria, the problem of overfitting and a selection bias of covariates with a maximum number of possible splits as a result of the numerical optimization arises.

Instead of using traditional classification and regression trees (CART) or related tree algorithms like ID3 or C4.5, we applied a different approach embedded in the context of statistical inferential theory (see [29,33]). We used a conditional inference tree (CTREE) based on multiple permutation tests which combines tree based regression and statistical theory of conditional inference. Opposite to CART or C4.5, CTREE controls for selection bias using splits based on statistical inference and significance values. Permutation tests are implemented to guarantee a solid stopping criteria. Thus, our model overcomes typical problems of classical tree algorithms.

To verify our results and detected splitting variables, variable importance scores were calculated based on conditional random forests for each cost sector.

When comparing CTREE results to CART, in all cost sectors CART lead to more splits on the one hand, while pruning lead to no suitable trees. On the other hand, CART verified the findings of CTREE by showing identical nodes and/or grown tree structures. Furthermore, CTREE lead to theoretically reasonable splitting variables. Based on these findings as well as on calculated error terms and results achieved from the conditional random forests, our study emphasizes the superiority of the CTREE algorithm.

Our statistical analysis was based on a pre-imputed master data set provided by the data management of the MultiCare study group which had used the hot deck method and conditional means for imputation of missing values. Although we are aware of benefits resulting from multiple imputation algorithms, we agreed on using the master data set for the sake of consistency and because the proportion of missing values in the variables used for our analysis was very small. Nevertheless, tree-based algorithms can handle complete data as well as missing data usually assuming Missing Completely At Random (MCAR).

Patients with response-limitations due to medical reasons (blindness, deafness, dementia, etc.) as well as nursing home residents were excluded from the study sample. Therefore the impact of respective chronic conditions on health care costs could not be analyzed. Yet conditions associated with response-difficulties may strongly influence health care costs. For example, dementia is a very important and prevalent condition in the elderly associated with high health care costs. Dementia is often present in late stages of different diseases, such as PD, CI, chronic stroke and others. Future studies analyzing the impact of multimorbidity on health care costs should therefore consider surrogate responders for data collection in such response-limiting conditions.

## Conclusions

In elderly patients suffering from multiple chronic conditions, PD and cardiac insufficiency appear to be the chronic diseases most influential on total health care costs irrespective of the number and combination of other co-existing chronic conditions. Irrespective of any combination and number of co-occurring diseases, costs are significantly influenced by only a limited number of factors.

## Abbreviations

AID: Automatic interaction detection; CART: Classification and regression trees; CCI: Cardiac insufficiency; CHAID: Chi-square automatic interaction detectors; COPD: Chronic obstructive pulmonary disease; CTREE: Conditional inference trees; GP: General practitioner; PAOD: Peripheral artery occlusive disease; PD: Parkinson's disease.

## Competing interests

The authors declare that they have no competing interests.

## Authors’ contributions

HHK, ME and HL conceived and designed the analysis. BW and GS prepared the data for analysis. ME analyzed the data. HHK drafted the manuscript. HB, AF, JG, HHK, HL, WM, KM, SRH, IS, SW, HvdB and MS participated in study design and implementation. All authors read and approved the final manuscript.

## Author’s information

* Principal investigators

Hendrik van den Bussche and Martin Scherer

## Pre-publication history

The pre-publication history for this paper can be accessed here:

http://www.biomedcentral.com/1472-6963/13/219/prepub
